# Ectoparasite fauna of the lebranche mullet, *Mugil liza,* from a coastal lagoon in the Southwestern Atlantic

**DOI:** 10.1590/S1984-29612026029

**Published:** 2026-07-20

**Authors:** Laura Frantelmo Cavalheiro, Rayane Duarte, Marco Antonio Santos Silva, Rafael de Almeida Tubino, Michelle Daniele dos Santos-Clapp

**Affiliations:** 1 Universidade Federal Rural do Rio de Janeiro - UFRRJ, Instituto de Ciências Biológicas e da Saúde, Programa de Pós-graduação em Biologia Animal, Laboratório de Biologia e Ecologia de Parasitos, Seropédica, RJ, Brasil; 2 Instituto Oswaldo Cruz, Fundação Oswaldo Cruz, Fiocruz, Programa de Pós-graduação em Biodiversidade e Saúde, Laboratório de Helmintos Parasitos de Peixes, Rio de Janeiro, RJ, Brasil; 3 Universidade Federal Rural do Rio de Janeiro - UFRRJ, Instituto de Ciências Biológicas e da Saúde, Laboratório de Biologia e Ecologia de Parasitos, Graduação em Ciências Biológicas, Seropédica, RJ, Brasil; 4 Universidade Federal Rural do Rio de Janeiro - UFRRJ, Instituto de Ciências Biológicas e da Saúde, Laboratório de Biologia Pesqueira e Modelagem Trófica de Ecossistemas Marinhos, Departamento de Biologia Animal, Programa de Pós-graduação em Biologia Animal, Seropédica, RJ, Brasil; 5 Universidade Federal Rural do Rio de Janeiro - UFRRJ, Instituto de Ciências Biológicas e da Saúde, Departamento de Biologia Animal, Laboratório de Biologia e Ecologia de Parasitos, Seropédica, RJ, Brasil

**Keywords:** Copepoda, Myxozoa, Polyopisthocotyla, Maricá Lagoon, Rio de Janeiro State, Copepoda, Myxozoa, Polyopisthocotyla, Lagoa de Maricá, estado do Rio de Janeiro

## Abstract

Coastal lagoons are among the most productive aquatic ecosystems worldwide and are widely used by mugilid fishes. *Mugil liza* stands out as one of the main resources of lagoon fisheries in southern and southeastern Brazil. This study aimed to identify the species composing the ectoparasite fauna of *M. liza* from the Maricá Lagoon, Rio de Janeiro State, Brazil. A total of 44 specimens of *M. liza* were examined, of which 95.4% were parasitized by at least one ectoparasite species. Six ectoparasite species were recorded. The copepod *Ergasilus caraguatatubensis* was the most representative species, with prevalence (P) of 86.4%, followed by the copepods *Bomolochus nitidus* (P = 43.2%), *Caligus* sp. (P = 2.3%) and *Naobranchia lizae* (P = 2.3%). The polyopisthocotylan *Metamicrocotyla macracantha* showed prevalence of 18.2% and the myxozoan *Myxobolus episquamalis* presented a low prevalence (P = 2.3%). The ectoparasite fauna of *M. liza* exhibited diversity, comprising six distinct families, highlighting the host's gills as an important site for parasite diversity. The results expand the environmental distribution of *E. caraguatatubensis*, *B. nitidus, Caligus* sp., *N. lizae* and *M. macracantha* within Rio de Janeiro State, increasing the known occurrence sites of these parasites, including lagoon.

## Resumo

As lagoas costeiras estão entre os ecossistemas aquáticos mais produtivos do mundo, sendo amplamente utilizadas por mugilídeos. *Mugil liza* se destaca por ser um dos principais recursos da pesca lagunar na região sul e sudeste do Brasil. O objetivo deste estudo foi identificar as espécies componentes da fauna de ectoparasitos de *M. liza* na lagoa de Maricá, no estado do Rio de Janeiro. Foram examinados 44 espécimes de *M. liza*, dos quais 95,4% estavam parasitados por pelo menos uma espécie de ectoparasito. Foram registradas seis espécies de ectoparasitos. O copépode *Ergasilus caraguatatubensis* foi a espécie mais representativa, com prevalência (P) de 86,4%, seguido pelos copépodes *Bomolochus nitidus* (P = 43,2%), *Caligus* sp. (P = 2,3%) e *Naobranchia lizae* (P = 2,3%). O poliopistocotilídeo *Metamicrocotyla macracantha* apresentou prevalência de 18,2% e o mixozoário *Myxobolus episquamalis* apresentou baixa prevalência (P = 2,3%). A fauna ectoparasitária de *M. liza* apresentou diversidade, com seis famílias distintas, demonstrando como as brânquias deste hospedeiro abrigam uma fauna parasitária relevante. Os resultados gerados ampliam a distribuição ambiental de *E. caraguatatubensis*, *B. nitidus, Caligus* sp., *N. lizae* e *M. macracantha* dentro do estado do Rio de Janeiro, aumentando os locais de ocorrência desses parasitos, incluindo sistemas lagunares.

## Introduction

Coastal lagoons are among the most productive aquatic ecosystems worldwide (Pérez-Ruzafa et al., 2024). They are transitional environments between the continent and the sea, where important coastal biogeochemical processes occur, contributing to the maintenance of biodiversity and the regulation of life cycles of marine, estuarine, and freshwater species, while serving as feeding and nursery areas (Behera et al., 2020; Blaber, 2000; Pérez-Ruzafa et al., 2011, 2020). In addition, coastal lagoons provide several ecosystem services, including flood control, erosion prevention, water supply, and coastal protection (Cataudella et al., 2015; Newton et al., 2018).

Among the fish species that frequently use lagoon environments are the mugilids. The mullet *Mugil liza* Valenciennes, 1836 stands out as one of the main resources of lagoon fisheries in southern and southeastern Brazil (Souza et al., 2017). *Mugil liza* is a catadromous species that develops to adulthood in freshwater or brackish environments but reproduces and spawns in marine waters (Garbin et al., 2014). It is distributed along the western Atlantic coast, from Florida (USA), Bermuda, and the Bahamas, throughout the Caribbean Sea to Argentina (Albieri et al., 2010; Froese & Pauly, 2026). This species exhibits a detritivorous feeding habit, also consuming planktonic material (Lebreton et al., 2011; Aguirre-Pabon et al., 2022). In Rio de Janeiro State, artisanal fisheries are an important subsistence activity, serving as a source of income and food for fishing communities (Santos et al., 2018). According to the *Ministério de Pesca e Aquicultura*, mullets are among the most commercially exploited fish species in Brazil (Brasil, 2015).

Fish parasites represent a significant component of global biodiversity, playing key roles in trophic webs and ecological interactions in transitional environments such as lagoons and estuaries (Giari et al., 2022). The study of fish parasite fauna in these environments is essential for assessing socioeconomic impacts related to fisheries and resource use, as it may reveal environmental changes, influence the health and commercial value of economically important fish species, and serve as indicators of ecological changes in highly exploited coastal ecosystems (Newton et al., 2014; Giari et al., 2022). According to Vidal-Martínez & Wunderlich (2017), parasites are effective bioindicators of host condition and habitat quality. Parasite populations may increase or decrease depending on their sensitivity to environmental quality (Lafferty, 1997).

In Brazil, 68 parasite species have been reported infecting *M. liza*, of this 32 are ectoparasite species (Eiras et al., 2007, 2016; Mentz et al., 2016; Meira-Filho et al., 2017; Borges et al., 2018; Gueretz et al., 2018; Oliveira et al., 2019; Duarte et al., 2020; Silva et al., 2024). In this context, the purpose of the present study was to identify the ectoparasite species composing the parasite fauna of *M. liza* in Maricá Lagoon, Rio de Janeiro State, Brazil.

## Material and Methods

**Figure 1:**
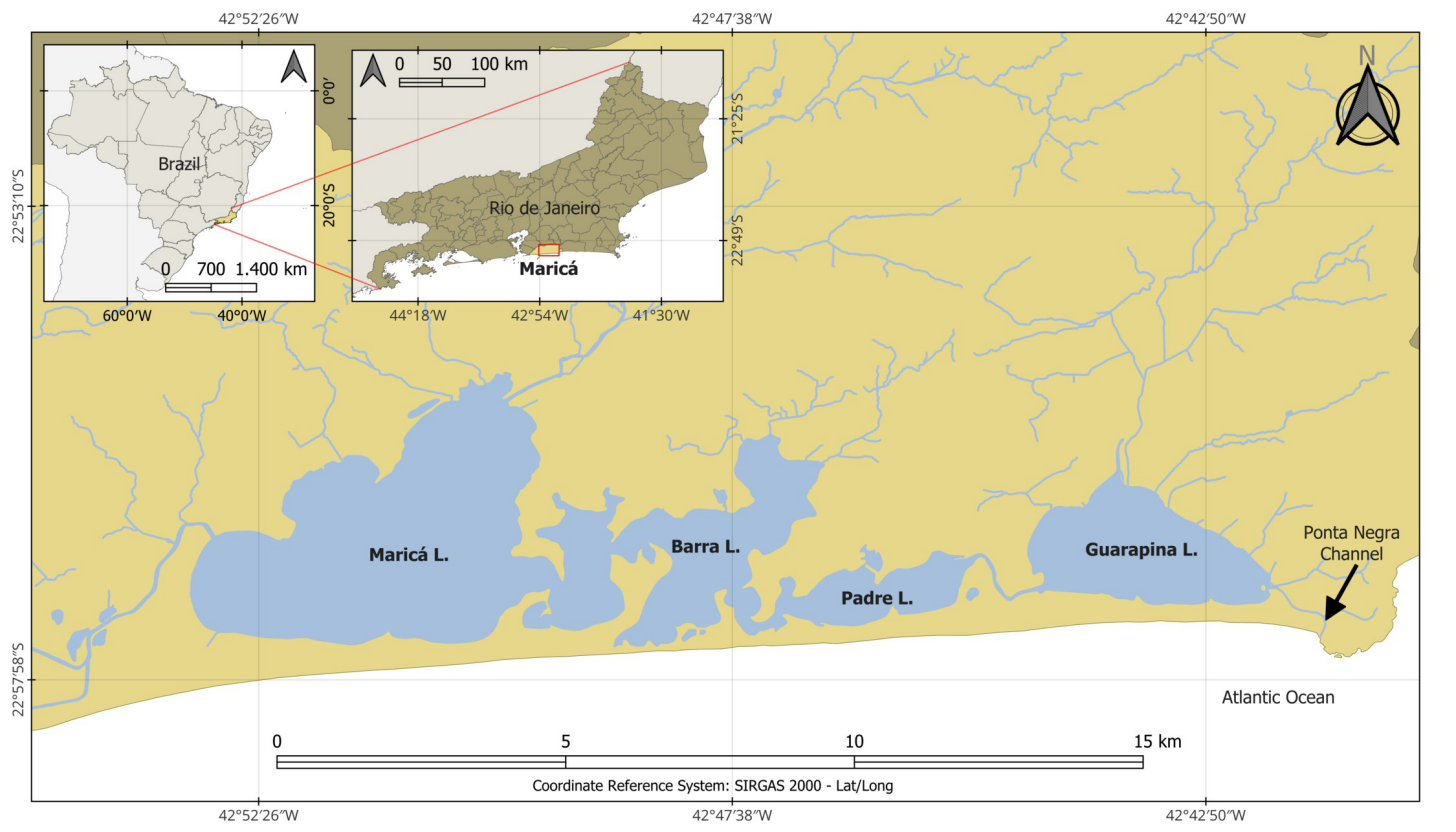
Maricá Lagoon, Rio de Janeiro State, Brazil, where specimens of *Mugil liza* were collected.

Between 2018 and 2023, 44 specimens of *M. liza* were collected from artisanal fishermen in the Maricá Lagoon (Figure 1), located in the municipality of Maricá, on the eastern coast of Rio de Janeiro State (22°56′41.1″ S, 42°51′09.7″ W). This lagoon is part of the Maricá–Guarapina Lagoon System, a predominantly brackish-water system (Dias et al., 2021) composed of interconnected lagoons that are also connected to the adjacent marine environment and represent one of the largest lagoon systems in the state (Toledo et al., 2021).

The fish were obtained fresh, immediately after capture. They were individually placed in plastic bags and stored in insulated containers with ice. Subsequently, the specimens were transported to the *Laboratório de Biologia e Ecologia de Parasitos* (LABEPAR), *Departamento de Biologia Animal, Instituto de Ciências Biológicas e da Saúde da Universidade Federal Rural do Rio de Janeiro*, where they were kept refrigerated or frozen until necropsy. Host identification was performed according to Menezes & Figueiredo (1980, 1985).

The fish were weighed using a digital scale. Sex was determined, and total and standard lengths were measured. All data were recorded on necropsy forms following the protocol proposed by Amato et al. (1991). During necropsy, the body surface was examined, and the gills were removed from the opercular cavity and individually placed in bottles. Gills from mullet specimens collected in 2018 and 2019 were fixed in 3% formalin, whereas those from fish collected in 2022 and 2023 were preserved in 70% ethanol. Subsequently, the bottles were vigorously shaken to promote detachment of parasites from the gill filaments. The gills were examined under a stereomicroscope using tweezers, stylets, and brushes. All ectoparasites recovered, except for representatives of Myxozoa, which were examined fresh, were preserved in 70% ethanol for subsequent identification.

Specimens of Myxozoa were classified according to Azevedo et al. (2010) and identified following Rothwell et al. (1997) and Eiras et al. (2006). Polyopisthocotyla were classified according to Brabec et al. (2023) and identified following Kohn et al. (1994). Copepoda species were classified according to Boxshall & Halsey (2004) and identified following Amado & Rocha (1995) and Eiras et al. (2016).

Parasitological descriptors (prevalence, intensity, and abundance), as well as the ecological terms used, followed the recommendations of Bush et al. (1997). Statistical analyses were performed on ectoparasite species with prevalence equal to or greater than 10%, according to Bush et al. (1990), using GraphPad Prism version 10.2.3. The significance level was set at *p*<0.05, following Zar (1999). Among the statistical tests, Spearman’s correlation coefficient by ranks (*rs*) and Pearson’s correlation coefficient (*r*), after prior angular transformation of the data (Sturges, 1926), were used to evaluate parasite intensity and abundance in relation to host size, and parasite prevalence in relation to host size classes estimated using Sturges’ formula, respectively. Parasitic dominance was calculated based on dominance frequency, shared dominance frequency, and mean relative dominance, following the method proposed by Rohde et al. (1995). The dispersion index (DI) was calculated for each identified species to determine the distribution pattern, and significance was tested using the *d* statistic (*d*>1.96), as proposed by Ludwig & Reynolds (1988).

Myxozoa cysts were deposited in the *Museu de Zoologia da Universidade Federal Rural do Rio de Janeiro* (MZUFRRJ). A representative specimen of Polyopisthocotyla was deposited in the *Coleção Helmintológica do Instituto Oswaldo Cruz* (CHIOC), and representative specimens of the Copepoda species were deposited in the *Coleção de Crustacea do Museu Nacional do Rio de Janeiro* (MNRJ-CARCINO). Accession numbers are provided in the results section.

## Results

The mean total length of *M. liza* from the Maricá Lagoon was 35.6±6.6 cm (26–52 cm), and the mean body weight was 435.0±238.9 g (178–1.208 g). Among the examined specimens, eight were males, and five were females. The remaining 31 hosts were classified as juveniles due to their smaller total length and the absence of gonadal development. Of the 44 mullets analyzed, 42 (95.4%) were parasitized by at least one species of ectoparasite. Six ectoparasite species were identified: *Ergasilus caraguatatubensis* Amado & Rocha, 1995, *Bomolochus nitidus* Wilson, 1911, *Caligus* sp., *Naobranchia lizae* (Krøyer, 1863), *Metamicrocotyla macracantha* (Alexander, 1954), and *Myxobolus episquamalis* Egusa, Maeno & Sorimachi, 1990. Among these, only *E. caraguatatubensis* was recorded throughout the entire sampling period, whereas the other species were found in fish collected only between 2018 and 2019.

## Parasite infracommunities

Among the ectoparasite fauna of *M. liza* from the Maricá Lagoon, *E. caraguatatubensis*, presented the highest parasitological indexes, followed by *B. nitidus*, and *M. macracantha*, as shown in Table 1. *Myxobolus episquamalis*, *Caligus* sp., and *N. lizae* showed prevalence values below 10% (Table 1).

**Table 1:** Occurrence of ectoparasites species of *Mugil liza* from Maricá Lagoon, RJ. Deposition numbers of voucher specimens (DN), prevalence (P), mean intensity (MI) and mean abundance (MA) and their respective standard deviation (SD), total number of specimens (TS) and site of infestation (SI) of the ectoparasites of *Mugil liza* from Maricá Lagoon, RJ, Brazil.

**Parasite species**	**DN**	**P (%)**	**MI ± SD**	**MA ± SD**	**TS**	**SI**
**Cnidaria**						
**Myxozoa**						
*Myxobolus episquamalis* Egusa, Maeno & Sorimachi, 1990	MZURM TZ2020021	2.3	N	N	N	CF, BS
**Platyhelminthes**						
**Polyopisthocotyla**						
*Metamicrocotyla macracantha* (Alexander, 1954)	CHIOC 39808	18.2	1.9±0.9	0.3±0.86	15	G
**Artropoda**						
**Copepoda**						
*Bomolochus nitidus* Wilson, 1911	MNRJ31875	43.2	5.5±3.9	3.2±3.9	105	G
*Caligus* sp.	MNRJ31877	2.3	1.0±0.1	0.03±0.1	1	G
*Ergasilus caraguatatubensis* Amado & Rocha, 1995	MNRJ 31876	86.4	12.9±18.1	11.1±18.1	490	G
*Naobranchia lizae* (Krøyer, 1863)	MNRJ31878	2.3	6.0±0.9	0.1±0.9	6	G

N = countless cysts, G = gills, CF = caudal fin, and BS: body surface.

The infestation sites recorded were the gills, with a total of 617 ectoparasites represented by five species (*E. caraguatatubensis*-490 specimens; *B. nitidus*-105; *M. macracantha*-15; *N. lizae*-six; and *Caligus* sp.-one specimen), as well as the body surface and caudal fin, which harbored innumerable specimens of *M. episquamalis*. These latter sites were infested exclusively by *M. episquamalis*, and due to the extremely high number of spores, the actual number of specimens could not be quantified.

Regarding dominance, *E. caraguatatubensis* showed the highest values of dominance frequency and mean relative dominance (Table 2).

**Table 2:** Frequency of dominance, shared frequency of dominance, and mean relative dominance of the components of the infracommunities of ectoparasites of *Mugil liza* from Maricá Lagoon, RJ, Brazil.

**Parasite species**	**Frequency of dominance**	**Shared Frequency of dominance**	**Mean relative dominance + Standard deviation**
*Metamicrocotyla macracantha*	1	0	0.043 ± 0.126
*Bomolochus nitidus*	11	1	0.244 ± 0.350
*Ergasilus caraguatatubensis*	29	1	0.667 ± 0.390

According to the dispersion index and the significant *d* statistic values presented in Table 3, the parasite infrapopulations recorded in this study exhibited an aggregated distribution pattern.

**Table 3:** Values of the dispersion index and the statistical *d*-test of the ectoparasites of *Mugil liza* from Maricá Lagoon, RJ, Brazil.

**Parasite species**	**Dispersion index**		
	**Value**	**Distribution**	***d***
*Metamicrocotyla macracantha*	0.74	Aggregate	4.45^*^
*Bomolochus nitidus*	6.57	Aggregate	14.55*
*Ergasilus caraguatatubensis*	29.56	Aggregate	41.20*

* significance level *d*>1.96.

The prevalence, intensity, and abundance of *B. nitidus* were higher in smaller fish, whereas the intensity and abundance of *E. caraguatatubensis* were higher in fish with greater total length (Table 4). Apparent negative correlations were observed between host size and the prevalence and abundance of *M. macracantha*, as well as between host size and the prevalence of *E. caraguatatubensis*; however, these relationships were not statistically significant (Table 4).

**Table 4:** Analysis of parasite indexes under possible influence of the total length of *Mugil liza* from Maricá Lagoon, RJ, Brazil.

**Parasite species**	**Prevalence**		**Intensity**		**Abundance**	
	***r***	***p***	***rs***	***p***	***rs***	***p***
*Metamicrocotyla macracantha*	-0.559	0.192	0.178	0.669	-0.044	0.775
*Bomolochus nitidus*	-0.904	0.005*	-0,533	0.018^*^	-0.442	0.002*
*Ergasilus caraguatatubensis*	-0.171	0.714	0.449	0.004*	0.370	0.013*

*r =* Pearson’s correlation coefficient*; rs =* Spearman’s rank correlation coefficient*.*

* significance level *p*<0.05.

Statistical analyses by host sex were not performed because most *M. liza* specimens collected were juveniles, which precluded this type of analysis.

## Discussion

In the present study, *M. liza* exhibited an ectoparasite fauna composed of representatives of the phyla Cnidaria (subphylum Myxozoa), Platyhelminthes, and Arthropoda (subphylum Crustacea). The Myxozoa representative, *M. episquamalis*, was recorded parasitizing a single host. The low prevalence observed contrasts with the study by Vieira et al. (2022), in which two distinct species, *Myxobolus curemae* Vieira, Agostinho, Negrelli, Silva, Azevedo & Abdallah, 2022 and *Myxobolus maceioensis* Vieira, Agostinho, Negrelli, Silva, Azevedo & Abdallah, 2022, were reported with prevalence values of 27% and 40%, respectively, in *Mugil curema* Valenciennes, 1836 from São Paulo and Alagoas.

Myxozoans generally exhibit a life cycle characterized by host alternation, involving a definitive host and an intermediate host. The occurrence of *M. episquamalis* parasitizing *M. liza* suggests that this fish species acts as an intermediate host for this group in the Maricá Lagoon. The first record of *M. episquamalis* infecting *M. liza* in Brazil was reported by Duarte et al. (2020). This species has also been recorded in other fish hosts, such as *Mugil cephalus* Linnaeus, 1758, and *Chelon ramada* (Risso, 1827) in Egypt, and *Planiliza macrolepis* (Smith, 1846) in Thailand (Lin & Ho, 1997; Eissa et al., 2020).

*Metamicrocotyla macracantha*, the only species recorded representing the phylum Platyhelminthes, showed the third highest prevalence (18.2%) among the ectoparasites identified in this study, with 15 specimens distributed among eight fish and an infestation range of one to four parasites per host. *Metamicrocotyla macracantha* and *M. inoblita* Buhrnheim, 1970 have previously been reported in fish of the family Mugilidae (Kohn et al., 1994; Baptista-Farias et al., 1995; Baptista-Farias & Kohn, 1998; Cohen et al., 2004; Baptista-Farias & Cohen, 2005; Moutinho & Alves, 2014; Mentz et al., 2016) and can be distinguished mainly by body size, the size of the genital spines, the number of testes, and the size and number of clamps (Kohn et al., 1994; Mentz et al., 2016).

Studies indicate that *M. macracantha* also occurs in brackish systems and transitional zones between marine and freshwater environments, such as Charleston Harbor and estuaries in South Carolina, United States (Baker et al., 2005, 2008). In Brazil, *M. macracantha* has been recorded infecting *M. liza* along the coast of Rio de Janeiro State (Kohn et al., 1994; Baptista-Farias & Cohen, 2005), in the Marapendi Channel (Baptista-Farias et al., 1995; Cohen et al., 2004), and in the Tramandaí–Armazém Lagoon System (Mentz et al., 2016). Abiotic factors, such as temperature, may influence parasite population dynamics (Kirk et al., 2022), as higher temperatures tend to favor increased rates of embryonic development (Rawson, 1976; Atroch et al., 2026) and, consequently, population growth (Bychowsky, 1958). In the present study, *M. macracantha* specimens were recorded in mullets collected in January, during the summer, which may have contributed to the prevalence observed.

The class Copepoda was the most representative group in terms of both species’ richness and number of specimens within the ectoparasite fauna of *M. liza* from the Maricá Lagoon, being represented by four species (*B. nitidus*, *E. caraguatatubensis*, *Caligus* sp., and *N. lizae*). Porto et al. (2022) highlighted the high parasite diversity associated with this class, reporting more than 7.000 described species, of which approximately 3.000 are recorded parasitizing freshwater fishes. Regardless of other factors that may be associated with the representativeness observed in the present study, there appears to be a tendency for mullets to harbor a greater number of representatives of this group, both in terms of species richness and total number of specimens, even when collected in brackish environments.

*Bomolochus nitidus* was the second species to show high prevalence (43.2%) in mullets, with an infestation range of one to 14 specimens per host. This species has been frequently reported parasitizing the gills of fishes of the family Mugilidae, commonly occurring in association with *Ergasilus* spp. (Eiras et al., 2016; Golzio et al., 2017; Falkenberg et al., 2021). Although *B. nitidus* is typically associated with coastal marine environments, records of its occurrence in brackish and estuarine systems parasitizing *M. curema* (Golzio et al., 2017; Falkenberg et al., 2021) demonstrate its ability to persist in transitional environments between marine and freshwater environments, as observed in the present study. Additionally, this species has been reported infecting *Mugil platanus* Günther, 1880 (= *M. liza*) along the coast of Rio de Janeiro State (Knoff et al., 1994), reinforcing its affinity with mugilid hosts in coastal environments.

Ergasilidae is one of the copepod families most frequently reported parasitizing fishes (Montú & Boxshall, 2002; Al-Sahlany et al., 2024), with *Ergasilus* von Nordmann, 1832 representing the genus with the greatest species diversity. In Brazil, Couto et al. (2023) and Corrêa et al. (2024) inventoried 77 species of Copepoda distributed in 18 genera of Ergasilidae parasitizing fishes. Among these, *Ergasilus atafonensis* Amado & Rocha, 1995, *E. bahiensis* Amado & Rocha, 1995, *E. caraguatatubensis* Amado & Rocha, 1995, *E. lizae* (Krøyer, 1863), *E. longimanus* Krøyer, 1863, and *E. versicolor* Wilson, 1911 have been recorded infecting *M. liza* and *M. curema* (Knoff et al., 1994; Fonsêca et al., 2000; Golzio et al., 2017; Falkenberg et al., 2021).

*Ergasilus caraguatatubensis* was the only copepod species recorded in this study presenting high prevalence (86.4%), occurring in 38 of the 44 mullets examined, with an infestation range of one to 88 specimens per host. The occurrence of this parasite in hosts of the family Mugilidae corroborates previous observations reported by Fonsêca et al. (2000), Golzio et al. (2017), and Falkenberg et al. (2021). This species has been reported parasitizing *M. liza* along the coast of Rio de Janeiro State (Knoff et al., 1994) and in a fish-farming pond in Itamaracá, Pernambuco State (Fônseca et al., 2000).

The transmission of ectoparasites is favored by close contact between parasitized and non-parasitized hosts, a process particularly relevant in schooling fish such as mullets (Silva & Araújo, 2000). This behavior may have contributed to the high infestation levels of *E. caraguatatubensis* observed in *M. liza* from the Maricá Lagoon. Representatives of the family Ergasilidae are found across a wide range of salinity regimes, which may be associated with their high prevalence in fishes such as *M. liza*. During their ontogenetic migrations from estuarine waters to the open sea, mullets experience broad salinity fluctuations (Rosim et al., 2013; Al-Sahlany et al., 2024).

Parasites assigned to the family Caligidae are characterized by relatively large body size and are popularly known as “sea lice.” *Caligus* spp. cause economic losses to fisheries, mainly by inducing stress in their hosts and causing severe damage to the skin and epidermal tissue, as well as secondary infections (Saraiva et al., 2015). Bondad-Reantaso et al. (2005) further noted that species of this genus may affect host growth, fecundity, and survival, as their feeding activity results in skin lesions. Species of *Caligus* are widely distributed in lagoon systems and saline waters worldwide (Knoff et al., 1994; Fonsêca et al., 2000; Fajer-Ávila et al., 2006; Mentz et al., 2016; Öktener et al., 2024). In Brazil, *Caligus* species previously recorded parasitizing *M. liza* include *Caligus minimus* Otto, 1821 and *C. praetextus* Bere, 1936, both reported by Fonsêca et al. (2000) in a fish-farming pond in Itamaracá, Pernambuco State, and *C. bonito* Wilson, 1905 along the coast of Rio de Janeiro State (Knoff et al., 1994). Additionally, *Caligus* sp. was documented parasitizing *M. liza* in the Tramandaí–Armazém Lagoon System by Mentz et al. (2016). In the present study, a single female specimen identified as *Caligus* sp. was recovered from the gills of a mullet in the Maricá Lagoon. Identification at the species level was hampered by the presence of *Udonella* sp. specimens attached to its external surface, an epibiotic platyhelminth frequently associated with caligid copepods (Kabata, 1973; Soares et al., 2021), which compromised the morphological structures required for precise identification.

*Naobranchia lizae* has previously been reported in Brazil parasitizing *M. liza* in the Guandu River (Azevedo et al., 2010), as well as *M. platanus* (= *M. liza*) along the coast of Rio de Janeiro State, where it presented a prevalence of 28.7% in a study conducted by Knoff et al. (1994). In estuarine environments, Baker et al. (2005) recorded *N. lizae* with prevalence of 17% parasitizing *M. cephalus* in South Carolina, and Teemer et al. (2016) reported its occurrence in the same estuary and host in the United States. The low prevalence observed for this species (2.3%) in mullets from the Maricá Lagoon contrasts with previous records, suggesting spatial variation or the influence of specific environmental factors. There is a lack of Brazilian studies evaluating *N. lizae* tolerance to lagoon environments characterized by salinity fluctuations and other environmental variables. In a study conducted in Egypt with *Naobranchia cygniformes* Hesse, 1863 parasitizing *Boops boops* (Linnaeus, 1758), Zayed et al. (2023) found significant correlations with physical factors, being positive with high salinity and temperature, and negative with low pH. In the present study, physical factors were not assessed, which precludes determining whether such variables influenced the low prevalence of *N. lizae* observed in the Maricá Lagoon, although a potential relationship cannot be ruled out.

Due to the fact that this study was conducted over a period that included sampling in 2018 and 2019, an interruption in 2020 and 2021 due to the COVID-19 pandemic, and the resumption of sampling in 2022 and 2023, the temporal scale is considered a potential limitation of this work.

Regarding possible changes in the occurrence dynamics of the recorded ectoparasite species, the persistence of *E. caraguatatubensis*, with similar parasitic indices over time, suggests temporal stability. On the other hand, the occurrence of the other species only in the pre-pandemic period may indicate that their presence is occasional, a pattern frequently observed in parasite communities, in which a few species are central and persistent, while others occur sporadically (Poulin, 1993).

*Ergasilus caraguatatubensis*, *B. nitidus*, and *M. macracantha* exhibited an aggregated distribution pattern, which is typical and characterized by the concentration of most parasites in a small number of hosts, whereas the majority of individuals show low parasite loads or are uninfected, a pattern commonly observed in fish parasites (Zuben, 1997). This finding corroborates the observations of the present study, in which higher parasite intensities were restricted to few hosts, while most mullets exhibited lower infestation intensities. Additionally, Poulin (1993) related parasite dispersion to physical environmental parameters, as well as to genetic factors and host immunological and behavioral susceptibility. Although these factors were not specifically assessed in the present study, they cannot be ruled out as potential drivers, particularly given that the study area is a coastal lagoon with a complex ecosystem that remains poorly investigated.

In accordance with Vilella et al. (2002), host body length reflects age and represents one of the factors influencing the size of parasite infrapopulations. Thus, the negative, albeit non-significant, correlations observed between host size and the prevalence and abundance of *M. macracantha* indicate that this parasite was more common and abundant in smaller fish. Total fish length influenced the intensity and abundance of *E. caraguatatubensis*, with larger hosts showing higher parasitism levels, a pattern also reported by Knoff et al. (1997) for the congeneric species *E. lizae* and *E. versicolor* parasitizing mullets from Rio de Janeiro. *Bomolochus nitidus* showed significantly higher prevalence, intensity, and abundance in smaller fish, indicating a significant negative correlation. Lizama et al. (2007) suggested that adult hosts may develop immunity to certain parasites, which could explain the higher infestation levels of some ectoparasites in juvenile fish and the reduced or absent infestation in adults.

The records of *M. macracantha*, *B. nitidus*, *E. caraguatatubensis*, *Caligus* sp., and *N. lizae* parasitizing *M. liza* in the Maricá Lagoon are relevant, as they expand the known distribution of these ectoparasites previously recorded in this host in other localities, thereby contributing to studies on fish parasites in Brazil.

## Conclusions

The ectoparasite fauna of *M. liza* from Maricá Lagoon was composed of one species of Myxozoa, one of Polyopisthocotyla, and four of Copepoda. All recorded taxa have previously been reported parasitizing mugilids in Brazil, reinforcing their association with the host and their geographic distribution.

The occurrence of *M. liza* in Maricá Lagoon expands the environmental distribution of *M. macracantha*, *E. caraguatatubensis, B. nitidus*, *Caligus* sp., and *N. lizae* in the state of Rio de Janeiro, increasing the number of recorded localities, particularly in lagoon systems. Notably, *B. nitidus* and *N. lizae* are recorded for the first time in a lagoon system in Brazil, as they had previously been reported only in marine environments. Additionally, *M. macracantha*, *E. caraguatatubensis*, and *Caligus* sp. are recorded for the first time in a lagoon system in the state of Rio de Janeiro.

Furthermore, mullets were shown to act as intermediate hosts within the local trophic community, particularly for *M. episquamalis*, reinforcing their ecological role in host–parasite dynamics in transitional environments.

Overall, this study contributes to the understanding of the diversity and distribution of fish parasites in coastal lagoon ecosystems, which remain relatively understudied in Brazil.

## Data Availability

The data supporting the findings of this study are available from the corresponding authors upon reasonable request.
